# It is a matter of convenience: why welfare technologies have become domesticated in Swedish eldercare

**DOI:** 10.1186/s12913-024-11924-x

**Published:** 2024-12-10

**Authors:** Susanne Frennert, Katrin Skagert, Anna Williamsson

**Affiliations:** 1 Department of Design Sciences, Lund University, Lund, Sweden; 2 Division Digital Systems, RISE - Research Institutes of Sweden, Stockholm, Gothenburg, Sweden; 3 Division Digital Systems, RISE - Research Institutes of Sweden, Gothenburg, Sweden; 4 Department of Sociology and Work Science, University of Gothenburg, Gothenburg, Sweden

**Keywords:** Eldercare personnel, Sweden, Welfare technology, Convenience, Science and Technology Studies (STS)

## Abstract

**Background:**

The use of welfare technology is gaining ground in municipal eldercare and is increasingly being integrated into everyday routines. However, the meanings that eldercare personnel attach to welfare technology in the care of older recipients, and thus the domestication of welfare technology, remain largely underexplored. This study explores how eldercare personnel understand and ascribe meanings to welfare technologies in their daily work, with the aim of understanding their domestication.

**Methods:**

The empirical material comprised 181 photographs, each paired with corresponding text, from 61 participants across four municipalities in southern Sweden. The empirical material was thematically analysed, focusing on different categories of welfare technologies and their ascribed meanings. In our coding “convenience” and at times “inconvenience” were interpreted as recurrent patterns. Their repeated presence across various contexts and the meanings ascribed to different welfare technologies prompted deeper interpretive engagement, leading us to adopt it as a key theme. In the final step, the codes were synthesised through the lens of “convenience” to better understand the meanings participants attached to welfare technology in eldercare work.

**Results:**

The participants ascribed meanings to welfare technology that resonate with broader societal and cultural understandings of technological solutionism, while aligning with national policies promoting welfare technology as a means of supporting safety, activity and independence for older adults. Welfare technology was often understood as both convenient and an act of care. Our analysis uncovered different dimensions of “convenience”, which we labelled as: “remote surveillance convenience”, “logistics convenience”, “communication convenience”, “safety convenience”, “comforting convenience” and “activation convenience”. Yet, in some cases, welfare technology was also seen as a hindrance to care, being inconvenient due to its inflexibility, technical difficulties and the tendency to create duplicate tasks.

**Conclusion:**

This paper contributes to a deeper understanding of the domestication of welfare technology in eldercare. Our study found that eldercare personnel engage with and interpret welfare technologies by ascribing meanings related to perceived convenience — a concept not widely explored in this context. Welfare technologies were often seen as convenient substitutes for physical proximity and relational care, such as “remote surveillance convenience” through cameras and “comforting convenience” via robotic pets. However, convenience, while central to the participants’ experiences, should not be understood as inherently “good” or positive but as part of the domestication process, shaped by socio-technical contexts and the political economy of eldercare, which prioritises effectiveness and efficiency. By shedding light on these dynamics, our study examines how the domestication of welfare technology is shaped by and reinforces broader discourses of technological solutionism, raising questions about its long-term impact on care practices.

**Supplementary Information:**

The online version contains supplementary material available at 10.1186/s12913-024-11924-x.

## Introduction

Assistant nurses in eldercare constitute a substantial segment of the workforce, with a majority being female and a considerable proportion being non-native workers [8, 36]. Assistant nurses are at the forefront of caring for older adults, both in their homes and in specialised housing. Their responsibilities encompass physical tasks such as aiding older adults with personal hygiene and mobility, medical tasks such as administering medications and relational tasks involving social interaction, such as accompanying older adults on walks and engaging in social activities [36]. Consequently, the role of assistant nurses is physically, emotionally and cognitively demanding [20].

Despite the critical nature of assistant nurses’ work, eldercare work is frequently perceived as low-paid and low-status, characterised by limited autonomy and substantial workloads [36]. Concurrently, the demographic landscape is shifting due to an ageing population, with an increasing proportion of older adults relative to the working-age population [38]. This demographic transition, facilitated by advancements in medical science and supportive environments, is a positive development [41]. However, it also exacerbates pressures on eldercare systems, leading to heightened costs and challenges in recruiting sufficient eldercare personnel [35]. Furthermore, the work environment for assistant nurses has attracted significant scholarly attention due to high turnover rates and significant occurrences of burnout [39]. To address these issues, the integration of technology in eldercare has been promoted as a viable strategy [67]. In Sweden and other Nordic countries, such technology is commonly referred to as welfare technology [58, 59]. Although there is no universal definition of welfare technology, the Swedish National Board of Health and Welfare describes it as digital technology aimed at maintaining or increasing safety, activity, participation and independence for individuals with or at risk of disabilities [58, 59]. This definition primarily focuses on care recipients rather than eldercare personnel or assistant nurses. However, the prevailing political discourse in Sweden regarding welfare technology emphasises its potential to address the needs of an ageing population while also positively impacting the recruitment and retention of eldercare personnel [50]. Efficiency and effectiveness in the context of eldercare personnel and welfare technology imply that fewer hands or individuals are required to care for an increasing number of older adults due to the utilisation of welfare technology [35]. Nevertheless, previous research on welfare technology has highlighted challenges in meeting the political expectations associated with welfare technology. Working with welfare technology in eldercare often entails additional, and sometimes invisible tasks [21, 48], difficulties in transitioning from isolated projects to full-scale implementations [25], as well as resistance [43] and workarounds [10, 24]. Despite these challenges, all municipalities in Sweden have implemented welfare technologies to varying extents [50].

This paper aims to explore the meanings ascribed by eldercare personnel to the use of welfare technology as it becomes integrated into municipal care. Our objective is to contribute to the growing body of research on the domestication of welfare technology in eldercare, enhancing understanding of welfare technologies’ roles and implications [10, 26, 34, 37, 42, 47]. While the theory of domestication is often regarded as a bottom-up process by individual households [3, 27, 55, 57], it has also been applied outside domestic settings [29, 40, 60–62]. In this study, we examine how top-down municipal decisions shape the incorporation and normalisation of welfare technology. In this context, domestication refers to how welfare technology becomes embedded in the day-to-day practices of eldercare personnel and how they attribute meanings to welfare technology based on their lived experience. While the municipalities set the frameworks and conditions under which welfare technologies are implemented, eldercare personnel engage with, interpret and adapt welfare technologies in their day-to-day practices based on the meanings they ascribe to them. This process is shaped and reflective of the broader cultural and institutional context in which they operate. Thus, the domestication process of welfare technology is an ongoing, dynamic process that emerge from the interplay between structural conditions and individual experiences [3, 27].

The structure of this paper is as follows. First, we provide background on the organisation and working of Swedish eldercare, along with references supporting the argument for an ideological shift towards digitalisation, self-care and efficiency. Next, we review relevant prior research on welfare technology and present a theoretical section on the concept of convenience. We then describe our methodology and present our analysis and findings. Subsequently, we conclude with a discussion that situates our study within a broader context, reflecting on the implications for practice.

### Background and related work

In the Nordic countries, municipal healthcare and social services provide care for older adults in need of assistance [1]. Healthcare and social services, funded by the welfare system, are allocated based on current welfare policies. Although older adults contribute to the cost, the welfare system subsidies these services. The municipality determines the type and extent of healthcare and social services for each individual, ensuring they meet the needs of the older adults without exceeding what is necessary. The different needs of older adults and the layout of their homes creates a “non-standardised” work setting, meaning that standardised procedures are not always applicable [15].

According to Kovalainen [35], many national innovation policies utilise technology as a foundational element, integrating it with care services within the framework of innovation and business strategies. Sweden, for instance, aspires to be a global leader in supporting its older citizens through digitalisation and welfare technology [19]. Consequently, the eldercare sector is frequently the focus of new technological innovations (i.e., welfare technology) and ‘digital first’ is becoming a mantra [33, 50]. Thus, technology is portrayed as “promise and remedy” to the crisis of care [35]. Recent research indicates that welfare policies and technological initiatives are implemented without considering the impact on the working environment of assistant nurses and eldercare personnel [11]. A comparison of the work situation of assistant nurses in Sweden between 2005 and 2015 shows that their work situation has dramatically worsened [64], due to the need to provide services to a larger number of older adults has increased. Furthermore, assistant nurses perceive that they receive less support from supervisors and colleagues, have less time to discuss and solve difficult situations encountered in everyday work life, as well as have less autonomy in planning their time [64].

#### On welfare technology and eldercare

Regarding welfare technology and the pressure and workload of assistant nurses in eldercare, a study on the introduction of medication dispensers in eldercare shows that the implementation shifted eldercare personnels’ focus from care arrangements to technical support. Thus, the body and relational work shifted towards more cognitive tasks [34]. A study on the introduction of sensor-floor technology shows that assistant nurses perceived the technology as simultaneously increasing both security and insecurity at work, as it draws attention from the older adults to the technology itself and raises issues of privacy and security [26]. Similarly, a study of the introduction of a social alarm system at special housing for older adults shows that the assistant nurses perceived added complexity in their work due to receiving alarms from residents, checking alarms via phones, responding to alarms via phones, checking specific residents’ situations in person and documenting all finished alarms [9, 10].

Rostad and Stokke’s [47] cross sectional survey in Norwegian municipalities reveal that 96% of home care services and 81.9% of nursing homes have either social alarms, fall detectors, bed and chair sensors or digital supervision [47]. Their study highlights that “the promise and remedy” of welfare technology in eldercare has mainly been translated into practice in form of surveillance technologies [47]. In municipal strategy documents, welfare technology is often connected to organisational matters [50]. Implementation and utilisation of welfare technology is perceived as necessary to meet the organisational and municipal responsibilities of providing care for older adults in need, while simultaneously addressing the challenges of retaining and recruiting eldercare personnel [50]. Nickelsen & Abildgaard [42], shows that in national and local policy documents, robots for feeding are portrayed as a solution to the problem of stressed healthcare workers and the lack of staff [42]. In this regard, robots for feeding are a “technical fix” and this approach can be labelled as technological solutionism [68]. The technological imperative, which views technology as inevitable and necessary, is evident in municipal policy documents on welfare technology [50]. This perspective is also reflected in a comparative study on telecare robots and eldercare workers’ perceptions. The study shows that while in 2016 eldercare personnel perceived telecare robots as incompatible with their personal values and philosophy of care, by 2020 their perceptions had become more positive [66]. This change may be explained because people’s perceptions change as they become more familiar to the technology. Initial fear and resistance may be overcome when the technology becomes more familiar. It may also be explained by that people’s work practices change when new technologies are introduced [45]. Initially these changes may be perceived negatively if they require more work but over time the technology becomes normalised into the working practice as people acquire the skills and habits to use the technology. Through a process of negotiation and adaptation at individual, group and societal levels, the technology becomes domesticated and embedded into everyday working life [3, 27, 56, 62]. Thus, the domestication process, in turn, connects innovation and design to public framing and the broader cultural context [2, 5, 29].

#### On convenience and why convenience matters

Most research on convenience has been done within marketing and consumption, especially connected to online banking [7, 32, 53], online shopping [16, 31, 49] and food consumption [18, 22]. The research on convenience in this context of consumption argues that the greater the time people save, the greater the convenience is perceived [4, 13, 52]. They also argue that the perception of convenience is negatively influenced by the perception of cognitive, physical and emotional effort associated with a product or service [4, 51, 52].

According to *anthropologist* Oka [44], interpretations of convenience as” a culturally shaped heuristic behaviour within a larger doxa informing the judgment or calculation, unconsciously or consciously, used to assess utility and/or satisfaction gained and time, effort/energy, and other culturally defined constraints expended, in order to make a decision on an intended activity” (p. 190). This interpretation moves away from seeing convenience just as something that save or shift time but as a multidimensional construct [54]. As such, convenience as a concept, is not just about making life easier but is deeply intertwined with cultural, ethical and moral considerations. It involves an individual or - a group to assess the utility and satisfaction gained from an activity or practice against time, effort and other constraints needed to perform that activity or practice. Decision of convenience are made by selecting the option perceived as the least inconvenient among many. Thus, perceived convenience is not purely a rational decision, but rather daily acts shaped by social forces influenced by norms and values at local, regional, national and global levels [44].

In the context of healthcare perceived convenience has been identified, particularly exemplified by the case of C-sections [44]. The convenience of C-sections has been categorised as follows: procedural convenience, offering a more controlled and time-efficient option for hospitals compared to unpredictable vaginal births; economic convenience, where C-sections, though more costly for patients, are often more cost-effective for hospitals, especially in regions where individuals or insurers bear the cost; and patient convenience, allowing procedures to align with patients’ schedules, medical needs and emotional well-being [44].

When it comes to welfare technology research and the digital transformation of eldercare, to our knowledge, perceived convenience is never mentioned. We can think of four reasons why. First, in the context of care, perceived convenience may have negative connotations as it may be perceived as taking shortcuts, being lazy or putting own needs before the care recipients’ needs. Second, welfare technology is a Scandinavian concept, and we do not have a word similar to convenience in our language. Third, much research on welfare technology tends to be conducted within caring science, which often adopts a normative stance, or within computer science, which frequently takes a technocentric approach. Forth, convenience originates from consumption and market research, while welfare technology is related to healthcare research in which the concepts of effectiveness and efficiency are more commonly used.

While all three concepts (convenience, effectiveness and efficiency) are goal-oriented and focus on optimisation, the values they refer to are slightly different: effectiveness (achieving desired outcomes), efficiency (maximising output with minimal resource usage), and convenience (ease of use, accessibility and user experience). In relation to how eldercare personnel use welfare technology in everyday life and their experiences of it, we argue that convenience matters, as it reflects the meanings that eldercare personnel ascribe to welfare technology usage, meanings shaped by social forces influenced by norms and values at individual, group and societal levels. Furthermore, we argue that convenience plays a significant role in the domestication process, illustrating how and why welfare technologies become incorporated and normalised in municipal eldercare.

## Methods

The aim of this study was to explore the meanings that eldercare personnel attached to the use of welfare technology in four Swedish municipalities. The design was explorative and draws on photo elicitation, supplemented with short explanatory texts [17, 28]. The study was carried out in spring 2024.

Photo elicitation is a participatory and visual research method to get insights into the participants everyday worlds [28]. It is often used within an interview setting to help researchers identify dimensions to the research that the researchers had not considered but may be important dimensions for the participants [46]. The photographs can either be generated by the researchers to provoke the participant to interpret the photographs or generated by the participants to illustrate what they find important [12]. In the latter, the participants can be asked to take any kind of photographs, or they may have directions from the researcher on what to focus on. The photographs can be used in conjunction with oral or written interpretations and explanations [28].

### Data collection

The primary data sources for our study consisted of photographs, each accompanied by written interpretations and explanations provided by the participants. In total, 62 participants consented to take photographs, resulting in the generation of 181 images, all supplemented with explanatory texts.

Participant selection was facilitated by representatives in four municipalities, who referred potential eldercare units to us, from which we in our turn could recruit participants to the study. The inclusion criteria encompassed employees working with welfare technology in either home service or specialised housing for older adults. The researchers conducted an online introductory meeting with each unit’s personnel, i.e. the team of assistant nurses, care assistants and their closest (unit) manager, during which the project and methodology were presented. Following this meeting, the unit manager informed the researchers about the collective interest of the personnel to participate. Subsequently, the researchers organised an onsite briefing for personnel regarding the project, methodology and their voluntary participation, followed by each individual personnel’s decision to participate and signing of informed written consent. Participants were then instructed to photograph all welfare technologies utilised in their daily work, providing details including the technology’s type (e.g., app, robot, sensor), usage location (e.g., kitchen, outdoors, bathroom, bed), specific facilitated activity, intended purpose and their experiences with the technology, indicating whether it contributed positively or negatively to their work (participants’ instructions detailed in Appendix 1). Additionally, participants were instructed to avoid taking photographs that revealed sensitive information, such as the identities or names of care recipients. Written instructions for the photo elicitation process were provided and the participants signed informed consent forms. Furthermore, during the onsite briefing the participants completed a short background survey regarding their experiences with care work and welfare technology. The survey, detailed in Appendix 2, was developed for this study.

The photo elicitation phase lasted five months, during which participants were asked to take photographs and provide accompanying written explanatory texts of the welfare technologies they encountered in their daily work. Throughout the phase, participants received weekly reminders to take photographs.

### Analysis

The analysis of the empirical material involved both the photographs and their accompanying written explanatory texts, which were considered as two complementary forms of data. In the first step, the researchers individually reviewed each photograph alongside its corresponding text. Each photograph, together with its text, was categorised into technology types using an Excel sheet. Photographs and their descriptions that were not considered welfare technology were assigned to a separate category. Then, the researchers tabulated the number of technology types identified across all photographs.

Next, the written explanatory texts accompanying each photograph within each technology type were thematically analysed following Braun & Clarke’s steps for thematic analysis [6]. Step 1 involved becoming familiar with the data, considering both the photographs and their corresponding texts. During this familiarisation, two notable observations emerged: (1) eldercare personnel prioritised the eldercare recipients’ experiences of welfare technology over their own experiences, and (2) eldercare personnel predominantly portrayed positive views and narratives of welfare technology usage. Step 2 involved coding the photographs and their accompanying texts to identify patterns. One common pattern identified was convenience and occasionally the inconvenience of using welfare technology. Initially, we did not utilise codes closely related to convenience, but after exploring different concepts and theories, we settled on ‘convenience’. Step 3 involved grouping codes from the photographs and their accompanying descriptions, using the construct of convenience to make sense of the emerging patterns. Step 4 entailed reviewing and refining the themes, consolidating them into overarching dimensions of convenience and inconvenience. In Step 5, the themes were described to encapsulate the core concepts and named. The themes were prioritised according to their prominence, determined by the quantity of photographs and the accompanying written descriptions received for each theme.

## Findings

When ascribing meanings to the use of welfare technology in eldercare, the participants highlighted the benefits for eldercare recipients. The impact of welfare technology on work conditions was seldom directly mentioned; instead, their descriptions indirectly addressed the convenience of using welfare technology, but also, in some instances, the inconvenience. Below, we present a description of the participants based on the background survey they completed prior to taking and sending the photographs and the corresponding written descriptions to us. Subsequent, we present the photographs we received, categorised by technology types, followed by our we our interpretation and analysis of the different forms of convenience described by the participants. We draw on both the visual and textual data. Our interpretation aims to capture how eldercare personnel ascribe meanings to different welfare technologies, with the aim to understand their domestication. Verbatim quotations from the written descriptions are included to illustrate each theme.

### About the participants

Among the 62 eldercare workers deciding to participate in the study 27 participants were from home care, 28 participants were from nursing homes and 7 were from municipal healthcare. Among the 55 participants from home care or nursing homes, 51 were educated assistant nurses, and 4 had received other care assistant training. The 7 participants from municipal healthcare were registered specialist district nurses.

**Fig. 1:**
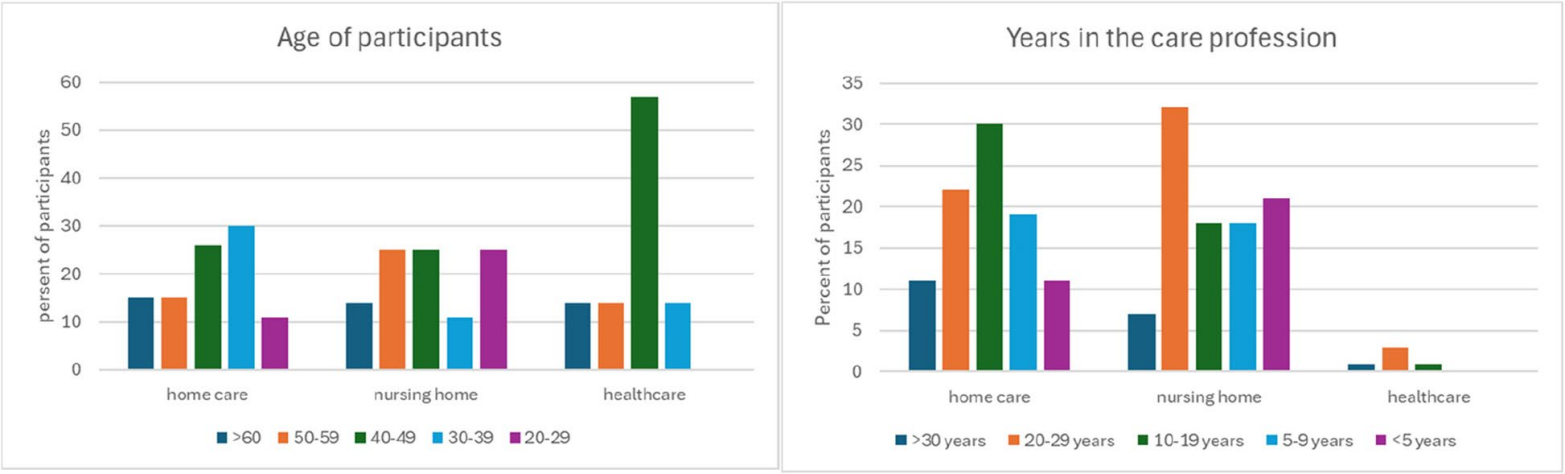
Distributions of participants’ age and years of experience in the care profession

Regarding employment conditions, all participants had fixed contracts where 77% of participants working full time (85–100% of 40 h/week) while 15% working part time (55–85% of 40 h/week) and 8% not answering the question. 26% of participants had another native language than Swedish. 8% of participants were men. Participants differed in age and amount of experience in the care profession, and both the youngest and oldest as well as most and least experienced personnel were found in nursing home teams (see Fig. 1).

### About the photographs – technology types

We received 181 photographs, each supplemented by a written explanatory text from the participants. Twenty-nine photographs that were not considered welfare technology were placed in a separate category. These photographs portrayed, among other things, electric cars, electric bicycles, lifts for transporting patients, electric wheelchairs and walkers. A total of 151 photographs with their respective descriptions were analysed and divided into the main themes of convenience and inconvenience (Table 1). Both the photographs and their corresponding description formed the core of our empirical material.

**Table 1:** Themes, subthemes, technology types, the number of photographs and corresponding written descriptions

**Themes**	**Subthemes**	**Technology types**	**Number of photographs **
**Convenience**	Remote supervision convenience	• door sensors • surveillance cameras • motion sensors • GPS tracking watches • personal emergency response systems	39
	Logistic convenience	• digital door locks/smart locks • mobile documentation apps	37
	Communication convenience	• digital care calendars • medical technologies, such as blood glucose monitor, blood pressure monitor, pulse oximeter, digital thermometer and digital scales	26
	Safety convenience	• smart lockable medicine cabinets • automated medication dispensers / medication dispensing robots	14
	Comfort convenience	• robotic cats, dogs and dolls, automated birds that chirp for the older adults • sensor-motor pillows	13
	Activation convenience	• consumer products such as smartphones, smart TVs • projector to engage the care recipients	9
**Inconvenience**	Double work inconvenience	◦ manual documentation at a desktop computer ◦ multiple, unsynchronised apps	5
	Inflexibility inconvenience	◦ food ordering via an app ◦ digital locks for the laundry room	4
	Technical problem inconvenience	◦ batteries that need to be replaced ◦ power outages ◦ noisy and inefficient robot vacuum cleaner	4

### Convenience as an act of care

The participants emphasised the benefits for care recipients of using welfare technology, highlighting its convenience in terms of remote supervision, logistics, safety, communication, comfort and activation. These conveniences were perceived as acts of care. According to the participants, welfare technology facilitated self-care and independence for eldercare recipients, as well as contributed to an enhanced care environment. The descriptions received from the participants portrayed welfare technology as not only convenient but also relational and interdependent, involving both caregivers and eldercare recipients within the context of use.

#### Remote supervision convenience

Through our analysis, we interpreted the theme of *Remote supervision convenience*, which reflects the perceived benefits among eldercare personnel of using welfare technologies that enable them to monitor care recipients from a distance, reducing the need for physical presence.

**Fig. 2:**
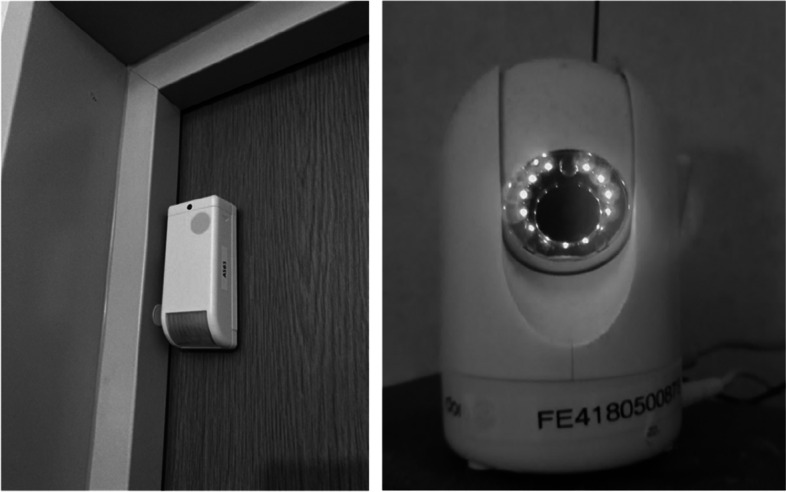
Example of participant photographs of a door sensor and a surveillance camera

According to the participants, welfare technology such as door sensors, surveillance cameras, motion sensors, GPS tracking watches and personal emergency response systems allowed them to monitor the older care recipients from a distance (see examples in Fig. 2). Participants frequently noted that the use of these types of welfare technologies provided reassurance by alerting them in case of emergencies or unusual behaviour without requiring constant direct supervision of the care recipients, interrupting the care recipients only when necessary. The participants expressed:

"*Good for us in our work so that we do not intrude when the older adult is sleeping or using the toilet.*"

"*Can be used to check that everything is OK with the older adult instead of physical visits, good for us to know if something has happened*."

"*Used as an aid to perform digital check-ins, detect falls, etc. It facilitates our work*."

These quotations highlight how care personnel found remote supervision useful in safeguarding care recipients while maintaining their privacy. The theme of welfare technology related to remote surveillance was particularly significant due to the highest number of photographs and corresponding written descriptions.

#### Logistic convenience

The theme of *Logistic convenience* was constructed based on participants’ frequent mentions of the ease and speed which eldercare personnel can access care recipients, obtain necessary information and document their actions using certain types of welfare technologies.

**Fig. 3:**
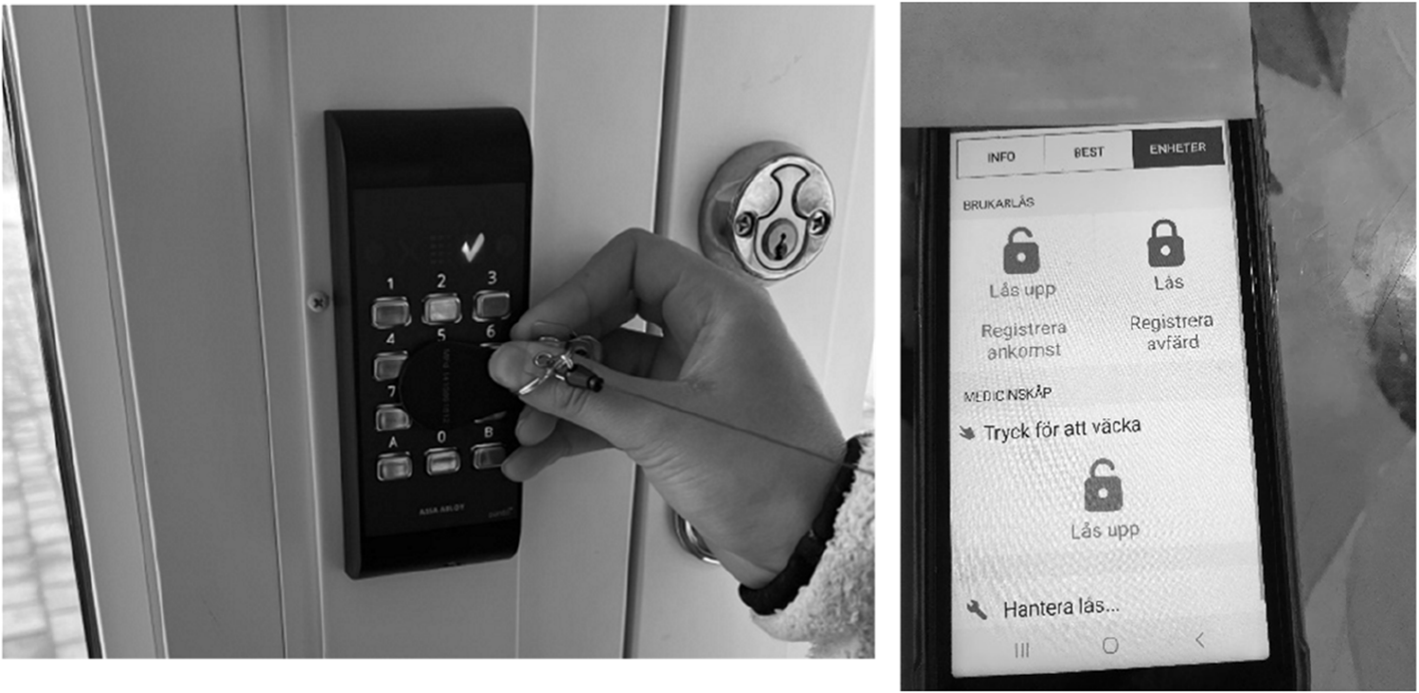
Example of participant photographs of a smart lock and an application to open doors

According to the participants, welfare technology such as digital door locks/smart locks and mobile documentation apps enhanced their efficiency by providing easy access to care recipients’ homes without the need for physical keys, as doors could be opened with a mobile phone (see examples in Fig. 3). Furthermore, through their mobile phones, they had access to information about the tasks that needed to be carried out and information about the care recipients’ preferences. The participants perceived that it was easy to document their actions via their mobile phones. Some participants referred to this as “mobile working,” which they appreciated for its ability to simplify and streamline their work. For example, one participant wrote:

"*We have this application that we use for time registration and opening doors with smart locks. It is very convenient because we have access to care recipients’ information when we are out and about. If we receive emergency alarms, we can directly enter the care recipients’ homes. It also helps us to know what to do and obtain information about each care receiver. We can also document what we do."*

This quotation exemplifies how care personnel found welfare technologies, such as smart locks and mobile apps, making their work more manageable and responsive.

#### Communication convenience

The theme of *Communication convenience* was interpreted from participants’ photographs and corresponding descriptions of how certain welfare technologies facilitated the exchange of essential information with care recipients.

**Fig. 4:**
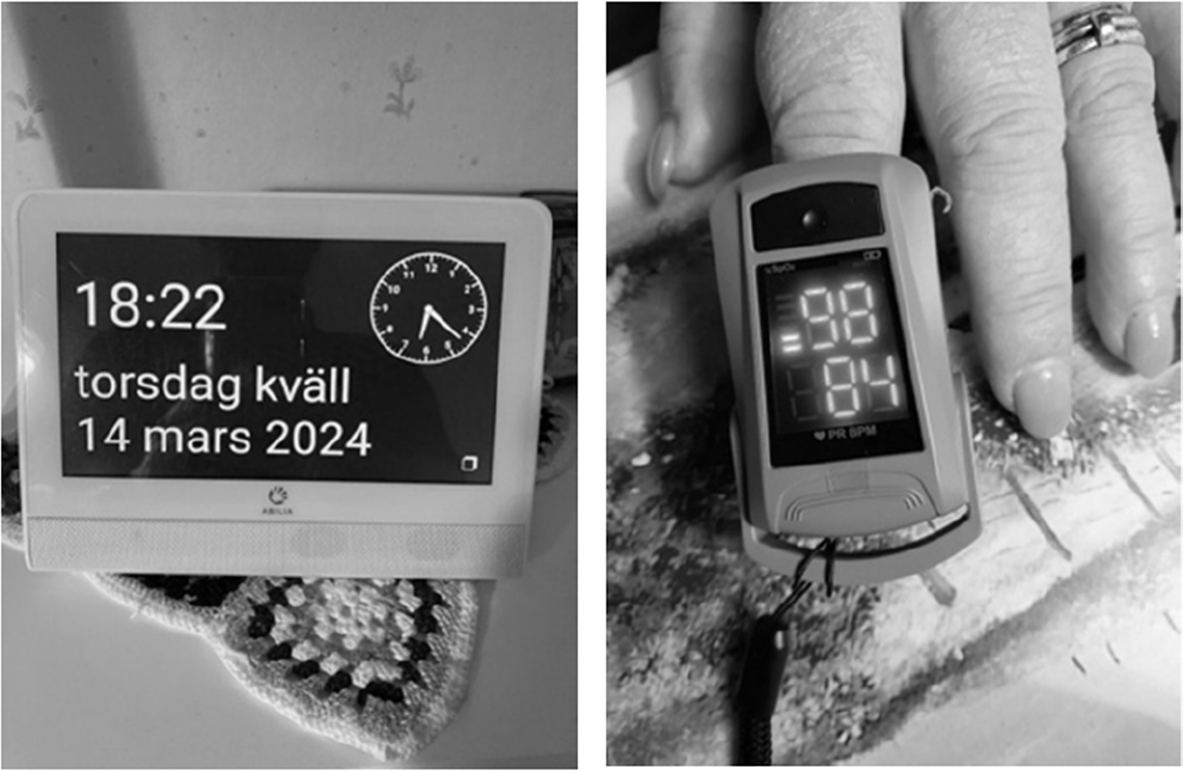
Example of participant photographs of a digital calendar and a medical technology device

In this context, communication convenience involved keeping care recipients up-to-date on necessary daily information and health parameters. Participants emphasised that digital calendars and medical technology, such as blood glucose monitors, blood pressure monitors, pulse oximeters, digital thermometers and digital scales, helped ease information exchange with care recipients (see examples in Fig. 4).

Digital calendars were particularly noted for their role in providing key information on the current day, time, month, planned activities and the weather forecast. As a result, eldercare personnel perceived that they did not have to repeatedly answer questions about the day, time and month. This eased their communication with care recipients and relieved them from having to repeat the same information over and over again. Some participants described that the digital calendars supported the care recipients to become more engaged in everyday life and less confused, which was perceived as reducing the workload for the eldercare personnel. As one participant noted:

"*Some care recipients are very confused. They ask what time of day it is and then again about what time of day it is. They mix up night and day. The digital calendar makes it easier for us. We don’t have to repeat the same thing all the time. It makes the care recipients more involved in their daily lives."*

This quotation illustrates how digital calendars not only provide important information to the care recipients but also ease the communication demands on eldercare personnel.

Additionally, participants highlighted the use of Medical Technology as easing communication with care recipients, as eldercare personnel could measure vital health parameters in the home and provide the results directly to care recipients. These vital health parameters could also be easily shared with colleagues via their smartphones.

#### Safety convenience

In our analysis, participants frequently ascribed the meaning of safety to welfare technologies such as medical robots and digital applications, a theme we refer to as *Safety convenience*. The functionality of some welfare technologies in promoting safety was highly valued by eldercare personnel. For instance, the locked medical cabinets, accessible via a smartphone application, were seen as particularly convenient, as participants no longer had to physically and daily deliver medication to care recipients. Instead, they only had to make sure that the care recipients got the right medication at the right time. Additionally, the use of a smartphone application to open the cabinets was perceived as enhancing safety, as it maintained a record of who accessed the cabinet and when. One participant, for example, described how the use of a smartphone application to access the medication cabinet enhanced safety:

"*This is good for the safety of care recipients, as many care recipients with dementia have lost their cognitive abilities and cannot manage when and how to take their medication. It can even become a great danger for care recipients if the medicine and tablets are left out as they may not remember whether they have taken them or not. We help the care receiver take the medication correctly and at the right time via our phone, where everything is specified in a special app that also allows us to open the medicine cabinet."*

Another example of safety convenience was the medical robots that automatically dispense medication at prescribed times. Participants believed that these robots ensured that patients received the correct medication at the right time. They also mentioned that the medication robot provides reminders for medication and could alert eldercare personnel if doses were not taken, which they believed further enhanced safety.

#### Comforting convenience

**Fig. 5:**
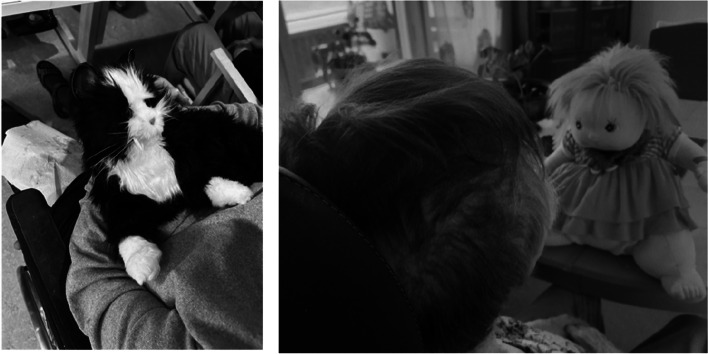
Example of participant photographs of a robotic cat and a robotic doll

We received photographs and corresponding written descriptions of robotic cats, dogs and dolls (see examples in Fig. 5), automated birds that twitter and sensor motor pillows, which constituted the theme *Comforting convenience*. Participants described how these welfare technologies were used to comfort agitated or anxious care recipients, making their work easier by keeping the care recipients calmer and more at ease.

Robotic cats and dogs, in particular, were described as relational tools for care recipients who felt lonely. According to the participants, the robotic pets provided companionship and gave care recipients a way to engage with the robots by caring for or brushing them. One participant illustrated this by saying:

"*We use robotic cats in special housing because they calm and engage the care recipients. They can take care of the cat and brush it. We get a calmer working environment."*

This quotation highlights how the use of robotic pets was ascribed the meaning of creating a more peaceful environment for both the care recipients and the eldercare personnel.

#### Activation convenience

**Fig. 6:**
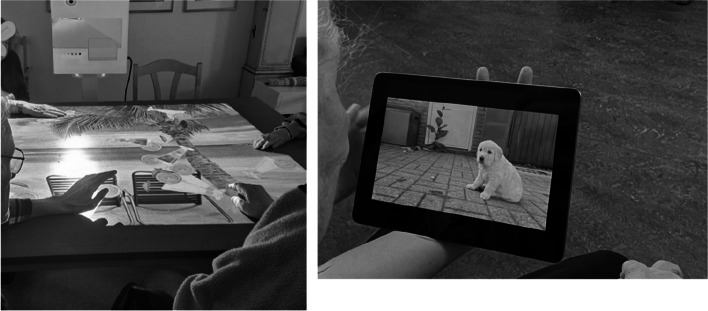
Example of participant photographs of activities stimulated by an interactive projector and the use of a tablet

The last theme of convenience, which we named *Activation convenience*, refers to welfare technologies or more generally to consumer products such as smartphones, smart TVs and interactive projectors (see examples in Fig. 6). These were described as convenient for activating the care recipients but also as easily accessible, offering a variety of choices when it comes to music and digital experiences that could be tailored to the care recipients. The participants described how consumer products could be used as a joint activity between the care recipients and caregivers to activate the care recipients but also as an activity to keep the care recipients busy and happy. As one participant noted:

"*When we use the interactive projector, our care recipients are usually happy and satisfied and then it becomes easier to help them."*

### Inconvenience as a hinder in the act of care

Although the participants mostly shared photographs and descriptions of the benefits for care recipients when eldercare personnel use welfare technology, a few participants highlighted the perceived inconveniences of using welfare technology, as seen in Table 1. Inconveniences such as double work, inflexibility and technical problems were perceived as a hinder in the act of care.

#### Double work inconvenience

The most common theme of inconvenience when working with welfare technology was the need to enter data both at the care receiver’s home on a mobile device and again on a stationary computer at the homecare office. A few participants shared photographs and corresponding descriptions of having to duplicate their work across different devices and switch between various applications, rather than of using a single application with multiple functions. They found this both time-consuming and monotonous. In some cases, they first wrote notes on paper at the care receiver’s home and then transferred the information onto a stationary computer at the office. In other instances, documentation had to be completed on both a smartphone and a stationary computer, further adding to the inconvenience.

#### Inflexibility inconvenience

The theme of *Inflexibility inconvenience* captures to the rigidity in the operation of certain welfare technologies and digital services, which participants described as a source of stress and additional, often invisible work. This theme was identified through the participants’ experiences with welfare technologies that disrupted their workflow, causing delays and inefficiency. One example shared by participants involved food deliveries ordered online. Participants highlighted how delays in deliveries, especially in bad weather, interrupted their workflow and caused frustrations. As one participant described:

*“Sometimes problems arise*,* for example*,* when the food deliveries are delayed*,* especially in bad weather. We struggle to wait because we only have 10 minutes to meet them and pick up the food for the care recipients. If the delivery is late*,* it disrupts our entire schedule.”*

This quote illustrates the frustration caused by delays, where participants had to adjust their time management to accommodate disruptions.

Another example of *inflexibility inconvenience* involved the use of digital locks in specific areas, such as the laundry room. Participants described that the locks required booking time slots in advance and if the eldercare personnel were late in picking up the washing, they could not access the laundry room. In such cases, they needed to contact their manager or landlord to gain access to the laundry room and sometimes they missed the opportunity to do the washing that week.

#### Technical problems inconvenience

The last theme of inconvenience, which we refer to as *Technical problems inconvenience*, relates to the technical issues participants had to address with welfare technologies. Overall, the robotic animals and dolls were described as providing companionship and joy for care recipients. However, participants also highlighted challenges. One participant mentioned that the robotic cats could become dirty and that certain welfare technologies required batteries that needed frequent replacement. For example, they had to ensure that GPS tracking watches were charged, which sometimes required a visit to the care receiver’s home just to recharge the devices. Another participant described a situation where one of the robotic dolls malfunctioned. She wrote:

*“In one instance*,* a user had become very attached to the robotic doll*,* carrying it everywhere. One day*,* the user was extremely upset and cried because she believed the robotic doll had ‘died’—it no longer opened its eyes or responded to her. The distressing experience spread to other users in the ward*,* causing anxiety. As a result*,* the situation became more challenging for the staff*,* as the malfunctioning robotic doll led the users to believe it had passed away.”*

This example illustrates how care recipients can become emotionally dependent on robotic dolls. When the doll malfunctioned, it disrupted these emotional bonds, leading to tension within the environment of the special housing facilities and placing additional pressure on the eldercare personnel. Eldercare personnel not only had to manage the technical issues with the robotic doll but also the emotional reactions that arose among the care recipients.

## Discussion

This study explores how welfare technology becomes integrated into everyday eldercare practices within Swedish municipalities by analysing the meanings that eldercare personnel attach to various welfare technologies. The findings suggests that while participants often associate welfare technology usage as both positive and convenient, these perceptions do not necessarily translate into tangible improvements in care quality or enhanced job satisfaction. Rather than treating convenience as given, we understand it as a dynamic experience, emerging within a specific socio-technical context, one shaped by broader shifts towards self-care, limited resources and an increasing focus on technological solutions. Perceived convenience, in this context, is not regarded as an inherent benefit of welfare technology, but rather as a set of meanings constructed by eldercare personnel as they navigate various systematic pressures. These pressures include the need to accomplish more with fewer resources, to rely on technological solutions and to emphasise efficiency.

In our data analysis, perceived convenience appears to manifest across several dimensions. For instance, technologies such as door sensors, surveillance cameras, motion sensors, GPS tracking watches and personal emergency response systems were perceived as enabling remote monitoring, thereby reducing the need for physical proximity to care recipients (which we refer to as “remote surveillance convenience”). Additionally, welfare technologies like digital door locks, smart locks and mobile documentation applications were perceived as enhancing the speed and ease with which eldercare personnel can access care recipients during emergencies, retrieve information and document their actions (i.e., “logistic convenience”). Furthermore, digital care calendars and medical technologies—such as blood glucose monitors, blood pressure monitors, pulse oximeters, digital thermometers and digital scales—were perceived as easing the communication of essential daily information and health parameters, thereby reducing repetitive exchanges and enabling timely updates (i.e., “communication convenience”). Safety was also perceived as improved through welfare technologies such as smart lockable medicine cabinets and automated medication dispensers or robots, which, according to the participants, ensured accurate medication handovers and thus enhanced the safety of medication administration (i.e., “safety convenience”). Moreover, robotic pets were perceived as comforting for care recipients, leading to a more tranquil working environment for the eldercare personnel (i.e., “comforting convenience”). Welfare technologies in the form of consumer products such as smartphones, smart televisions and projectors were perceived as engaging the care recipients in various activities, including videoconferencing with loved ones, watching specialized programs for people with dementia and supporting participation in training sessions (i.e., “activation convenience”). These findings contrast with past research, which suggested that welfare technology might face resistance from eldercare personnel due to concerns about role change, threats to professional identity and perceived challenges to fundamental care values [43, 69]. Contrary to these concerns, our study suggests that eldercare personnel often view welfare technology as facilitating self-care and promoting independence among care recipients, while also contributing to an improved care environment. Our analysis suggests that convenience is a contingent, context-specific experience, one that reflects the broader restructuring of care work in an age of technological solutionism and resource scarcity.

When it comes to robotic pets, our findings align with other studies [30, 65] on how robotic pets were found to reduce agitation among care recipients, thereby enhancing the work environment for eldercare personnel. In 2006, Sparrow and Sparrow published a well-cited paper in which they critique the use of robots in eldercare [63]. Sparrow and Sparrow argue that it is not only misguided to believe that robots can offer care and companionship to older people, but also unethical, as it involves deceiving older people and can lead to dehumanized care. In our study, the eldercare personnel did not raise any of these critiques towards robotic pets, nor did they express concerns that remote surveillance might reduce human contact and relational work. Interestingly, the participants also did not express any concern about themselves being monitored via their mobile phones or the oversight by managers in tracking their activities. Instead, they emphasised the benefits of welfare technologies for older care recipients, using words such as “increasing safety,” “activity,” “participation” and “independence” to describe the perceived experiences of welfare technology usage. These concepts can be traced back to “the promise and remedy” of welfare technology in policy documents and municipal strategies [14]. According to Foucault, the discourse in policy documents is a means of exerting power and control over what kinds of knowledge are considered normal and acceptable [23].

Our findings suggest that the discourse and ideas from policy documents or management about the advantages of welfare technologies for older care recipients have become internalised by the eldercare personnel, influencing how they think and describe the usage of welfare technology in their everyday work life. Their descriptions were almost solely focused on the benefits to care recipients when using welfare technology, even though they had been asked to describe how welfare technology affected their work. Their descriptions did not relate to physical tasks but rather to administrative tasks, such as administering medications that could be handled by welfare technology applications and relational tasks that could be handled by robotic pets and dolls, smart TVs and projectors. Another explanation for our findings may be that they reflect the current work situation of eldercare personnel, which is often described as time-constrained and stressed [20, 64]. The perceived convenience of using welfare technology among our participants might, therefore, be derived from the increased pressure and workload faced by eldercare personnel. For example, ‘remote surveillance convenience’ meant a reduced need for physical proximity to the care recipients and ‘comforting convenience’ meant that robotic pets and dolls comforted the care recipients and kept them calm, perhaps during times when the eldercare personnel could spend time on other tasks rather than comforting and calming the care recipients. It is somewhat worrying that our findings indicate that the use of welfare technologies are often perceived as convenient and as substitutes for physical proximity and relational work, without eldercare personnel reflecting on how this affects their work and job satisfaction. Evidence from past research suggests that the satisfaction derived from eldercare work is largely influenced by the relationships formed with care recipients and the perceived positive changes in the care recipients’ lives [20, 64].

An alternative explanation for our results could be that our methods did not capture critiques. However, we used a bottom-up approach in which the participants themselves choose which welfare technologies to photograph and to describe their experiences in their own words [17, 28]. Our hope is that the method provided an unbiased account of the meanings eldercare personnel ascribe to the use of welfare technologies in their everyday work life. Moreover, while the participants did not express resistance or critique of welfare technology usage, some mentioned the inconveniences that occasionally occurred, such as inflexibility, technical problems and double work, giving a somewhat nuanced view of how welfare technology affects their work. These issues sometimes required them to adapt their pace of work to the welfare technology or service in question (referred to as “inflexibility inconvenience”), resolve technical problems (“technical problems inconvenience”) and document the same things twice in different devices (“double work inconvenience”).

Another limitation is that we cannot determine, based on our results, how welfare technology affects job satisfaction. One may speculate that the use of welfare technology could reduce the relational aspects of care work, while increasing surveillance and digital monitoring, which, in turn, may lead to a loss of meaningfulness and job satisfaction. Based on the responses of our study’s participants, we can conclude that welfare technology is mainly perceived as convenient, with minimal resistance to its adoption. Thus, in our analysis of the domestication of welfare technologies, we interpret the meanings that eldercare personnel attach to welfare technologies as an example of how such meanings are co-produced through social, political and technological changes. Welfare technologies are integrated into eldercare in response to personnel shortage, time constrains and resource limitations. However, they also seem to carry implicit assumptions about what care might look like. The themes “remote surveillance convenience” and “logistic convenience” may be understood within the context of neoliberal policies that prioritise cost-effectiveness, efficiency and self-care. Consequently, care and care work may become reframed as areas where optimisation through technological solutions is possible. For eldercare personnel, welfare technology appears to have the potential to reconfigure both the nature of their work and their role within the socio-technical system of eldercare. This raises interesting questions about the ways in which welfare technology might impact job satisfaction. Future research may explore the connection between job satisfaction and the use of welfare technology.

## Conclusion

In conclusion, our analysis shows that eldercare professionals frequently ascribed perceived convenience to the use of welfare technology. The concept of perceived convenience attributed to welfare technology should be understood not as an intrinsic positive characteristic, but as a set of meanings shaped by eldercare personnel as they navigate high care demands and limited resources. In our analysis, perceived convenience appears to unfold along multiple dimensions: “remote surveillance convenience,” where welfare technologies allowed personnel to supervise care recipients from a distance; “logistic convenience,” which facilitated the rapid and easy access to care recipients during emergencies, as well as the retrieval of information and documentation of actions; “communication convenience,” where welfare technologies simplified the exchange of essential daily information and health parameters, reducing repetitive communication and enabling timely updates; “safety convenience,” which allowed for the controlled access to medication for certain personnel and care recipients at specific times; “comforting convenience,” where welfare technologies contributed to the comfort of care recipients, leading to a calmer working environment; and “activation convenience,” where welfare technologies supported the activation and engagement of care recipients. However, the study also revealed that in a few instances, welfare technology was perceived as inconvenient, primarily due to its inflexibility, technical difficulties and the potential for duplicating work. For eldercare personnel, welfare technologies shape their daily work within an evolving socio-technical system. The sense of convenience experienced by eldercare professionals reflects the broader ideological, political and social shifts influencing care practices and technological developments. The findings of the study suggest that the experience of convenience in the use of welfare technologies, rather than being inherently positive or negative, is a construct shaped by the interplay of daily actions, care practices, technological solutionism, and the wider social and political context.

## Supplementary Information

Supplementary Material 1.

Supplementary Material 2.

## Data Availability

The full dataset produced and analysed during this study are not publicly available in order to safeguard participant anonymity. However, they can be provided in Swedish by the corresponding author upon reasonable request.
