# Supplementary material for: It is a matter of convenience: why welfare technologies have become domesticated in Swedish eldercare
Source: BMC Health Serv Res. 2024 Dec 10;24:1558. doi: 10.1186/s12913-024-11924-x (PMC11629506; doi:10.1186/s12913-024-11924-x)
Supplement: Supplementary file 1 — Supplementary Material 1. [file 12913_2024_11924_MOESM1_ESM.docx]

# Photo instructions to eldercare teams

You are asked to take about 10 photographs over a three-week period. You take photos using your mobile camera. The photographs must be of different situations during your working day where different types of welfare technology are used. It can be, for example, medication dispenser, GPS alarm, apps for memory training or communication and more.

You choose which situations you want to photograph, but we would like you to photograph situations, events or objects that show your experiences of using welfare technology in your work with clients/users/elderly.

For ethical reasons, it is important not to offend or reveal the identity of any user when taking photographs. Therefore, here is advice and tips on what you should consider when taking photographs in work situations where welfare technology is used. Read this entire instruction manual before you start shooting.

Avoid taking photos of…

- ... client’s/user's/relative's/colleague's face
- ... client’s/user's private items that could reveal the client’s/user's identity (e.g. medicines or aids marked with name or social security number, driver's license or other identification)
- ... intimate situations, body parts or parts of the home (e.g., going to the toilet or showering/washing, too much bare skin, dirty laundry, toilet/bathroom)

Try to photograph…

- ... the actual situation and the use of welfare technology
- ... a situation when you yourself use some form of welfare technology (enlist the help of a colleague or user who takes the photo)
- … a situation when a user uses some kind of welfare technology in a real situation (considering what you should avoid according to above)
- ... a situation when a colleague uses some kind of welfare technology together with a user (considering what you should avoid according to above)

Keep in mind that you should…

- … try to include details in the photo that provide as much information as possible about the situation in which the technology is used
- ... turn on the lighting if possible so that the photograph is bright enough and as clear as possible
- … photograph one type of welfare technology in one photo and another type of welfare technology in another photo
- … photograph a variety of photos that show both positive and negative sides of using welfare technology (it can also be both positive and negative sides of the same welfare technology)

When you have photographed, we want you to briefly describe what is shown in each photo.

Include in the following points:

1. What kind of technology it is or what you call it (type of app, robot, sensor or similar)
2. Where the technology is used, e.g. in the kitchen, outdoors, in the bathroom, in bed
3. The activity in which the technology is used, and for what purpose it is used
4. How you or the user experience the use, e.g. whether it contributes to something positive or negative in the meeting with the user

Use the points in this guide as a checklist to go through before sending your photographs. Send the photographs together with the short description of your thoughts or experiences via SMS to researcher XX, mobile number XXXXXXXXXX.

Good luck!
