# Supplementary material for: It is a matter of convenience: why welfare technologies have become domesticated in Swedish eldercare
Source: BMC Health Serv Res. 2024 Dec 10;24:1558. doi: 10.1186/s12913-024-11924-x (PMC11629506; doi:10.1186/s12913-024-11924-x)
Supplement: Supplementary file 2 — Supplementary Material 2. [file 12913_2024_11924_MOESM2_ESM.pdf]

Background questions

First name: Surname:
Occupation: Municipality:
Work email:

Do you have any healthcare or other professional training?

1 ☐ Yes, care assistant training
2 ☐ Yes, assistand nurse training
3 ☐ Yes, other secondary or higher education
4 ☐ No

How many years have you worked in a caregiving profession?

1 ☐ 30 years or more
2 ☐ 20–29 years
3 ☐ 10–19 years
4 ☐ 5–9 years
5 ☐ Less than 5 years

What type of employment do you currently have?

1 ☐ Permanent employment
2 ☐ Temporary employment

What is your working hours commitment?

1 ☐ Full-time (85–100%)
2 ☐ Part-time (55–84%)
3 ☐ Half-time (ca 50%)
4 ☐ Less than half-time (5–45%)

In which field do you work?

1 ☐ Home care/ ordinary housing
2 ☐ Special housing
3 ☐ Home healthcare

What do you identify as?

1 ☐ Women
2 ☐ Man
3 ☐ Other gender identity
4 ☐ Prefer not to

Where were you born?

1 ☐ In Sweden
2 ☐ In another Nordic country
3 ☐ In another European country
4 ☐ In a country outside Europe

What is your age?

1 ☐ 60 years or more
2 ☐ 50–59 years
3 ☐ 40–49 years
4 ☐ 30–39 years
5 ☐ 20–29 years
6 ☐ 18 – 20 years
7 ☐ Younger than 18 years

What is your mother tongue?

1 ☐ Swedish
2 ☐ Other: Please specify:

Thank you for your participation!
